# An unexpected presentation of a traumatic wound on the lower lip: a case report

**DOI:** 10.1186/1752-1947-8-298

**Published:** 2014-09-07

**Authors:** Jaume Miranda-Rius, Lluís Brunet-Llobet, Eduard Lahor-Soler, Carlos Mendieta

**Affiliations:** 1 Departament d’Odontostomatologia, Facultat d’Odontologia, Universitat de Barcelona, Barcelona, Spain; 2 Servei d’Odontologia, Hospital Universitari Sant Joan de Déu, Universitat de Barcelona, Barcelona, Spain

**Keywords:** Human bite, Lip injury, Orofacial wound

## Abstract

**Introduction:**

Traumatic lip injuries present major challenges in terms of reconstructive options and the outcome of surgical management. The aetiology of lip injuries includes human bite as interpersonal violence. Bite wounds are always considered to be complex injuries contaminated with unique polymicrobial inoculum. A classification of facial bite injuries has been included and the surgical management of these lesions has also been discussed. We report a rare bite injury on the lower lip that resembled an ulcerative process.

**Case presentation:**

A 30-year-old African man presented with a severe tissue defect on his lower lip to a Dental and Oral Department in Tanzania. He explained that 12 days ago he had been involved in a fight and someone had bitten his lower lip. An orofacial examination confirmed a serious loss of lip tissue that resembled a chronic ulcerative process. Accurate assessment of the lesion was made by a thorough evaluation of some parameters such as size, depth, presence of granulation tissue, fibrin coverage, wound edges, exudates and/or necrosis. A surgical debridement under local anaesthesia was carried out. Afterwards a layered suture was performed. Eventually the healing was complete and satisfactory.

**Conclusions:**

A severe bite avulsive wound on the lower lip, despite the elapsed time before treatment, may have an excellent prognosis after a simple surgical procedure.

## Introduction

Traumatic lip injuries present major challenges in terms of reconstructive options and the outcome of surgical management [1]. The aetiology of lip injuries varies, depending on socio-environmental factors. These include road traffic accidents, human bite, animal bite, burns and electrical injuries, as well as interpersonal violence [1-3]. Commonly, human bites by an assailant occur extra-orally but on a few occasions they also occur intra-orally. The size and severity of the injuries vary, ranging from small lacerations, punctures or cuts to total avulsion and loss of relatively big chunks of tissue [4]. Because of their location human bites in the maxillofacial region compromise function and aesthetics; as a consequence social and psychological effects are most likely [5].

The prevalence of human and animal bite injuries is around 1% of all emergency injuries. The frequency by affected areas, varies from cervicofacial region (36%), lower extremity (31%), upper extremity (19%) and chest (14%) [5].

Bite wounds are always considered to be complex injuries contaminated with unique polymicrobial inoculum. Human bites are as serious as animal bites because they induce a higher incidence of infectious complications. Human saliva is considered to be a virulent inoculum, with bacterial loads at the order of 10^8^ per millimetre, which significantly increase in cases of periodontal disease and oral sepsis [6].

In bite wounds to the face, infectious complications can create more difficulties than the initial tissue damage itself for the task of restoring an aesthetic appearance. The management should aim to neutralize this potential for infection and provide an infection-free environment for wound healing [6].

An atypical orofacial traumatic wound type IIIA, according to Lackmann’s facial bite injuries classification, is presented. The appearance of the lesion because of the elapsed time between the trauma and the patient’s attendance adds interest.

## Case presentation

**Figure 1:**
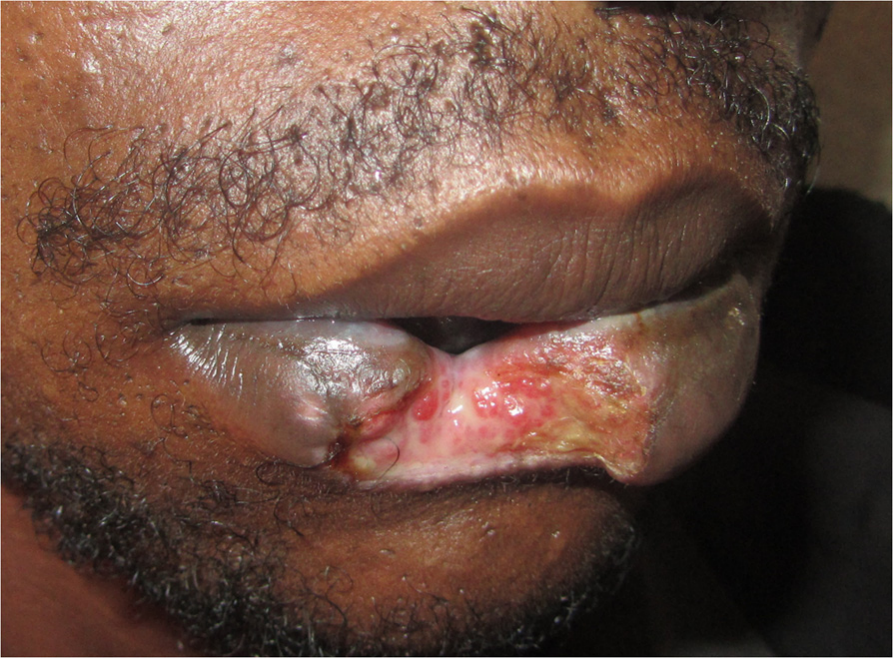
**Clinical image: A human bite injury.** Notice the severe lip tissue defect mimicking an ulcerative chronic process.

A 30-year-old African man presented with a severe tissue defect on his lower lip (Figure 1) to a Dental and Oral Department in Tanzania. He had no past medical history of note.

**Figure 2:**
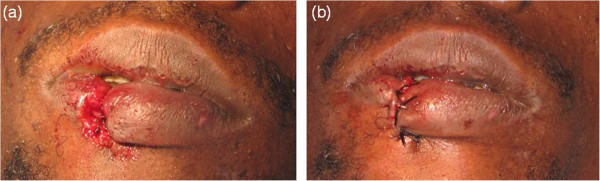
**Surgical procedure. (a)** and **(b)**. After debridement a layered suture was performed. Notice vertical mattress stitches through the muscular-subcutaneous tissue and through the skin in order to get a stable primary wound closure.

**Figure 3:**
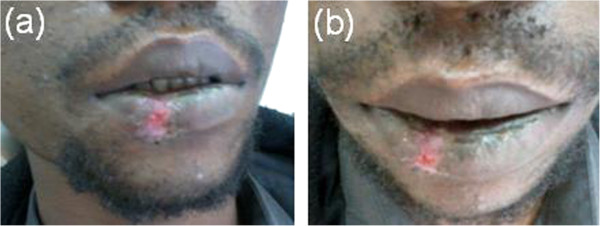
**Postoperative images. (a)** and **(b)**. After removing sutures.

He explained, while covering his mouth with a scarf, that 12 days ago he had been involved in a fight and someone had bitten his lower lip. After initial emergency first aid and several topic cures at a local dispensary, he was referred to the regional hospital. An orofacial examination confirmed a serious loss of lip tissue that resembled a chronic ulcerative process. Accurate assessment of the lesion was made by a thorough evaluation of some parameters such as size, depth, presence of granulation tissue, fibrin coverage, wound edges, exudates and/or necrosis.A surgical debridement and manual irrigation with normal saline under local anaesthesia was carried out. Afterwards a layered suture was performed with what we only had available at that service, catgut for muscular/subcutaneous tissues and silk for skin/oral mucosa. There was some over tension around the vertical mattress sutures as it was important to get a correct primary wound closure (Figure 2). Postoperative medical treatment included oral analgesics (ibuprofen 400mg 8 hourly for 5 days) and broad spectrum oral antibiotics coverage (amoxicillin 500mg 8 hourly for 5 days in combination with metronidazole 500mg 8 hourly for 5 days). In order to avoid a possible cicatrization failure, silk sutures were removed after 2 weeks. Eventually the healing was complete and satisfactory (Figure 3).

## Discussion

Most lip defects occur as a result of trauma, infection (cancrum oris), burns or following a surgical excision of lesions [1,2,5,7]. However, in agreement with the vast majority of African studies, the most common aetiology of lip injury is human bite.

The lower lip is the site most commonly involved, followed by the ear. This could be explained by the position of the lower lip in the face, which makes it among the most prominent parts in the face and therefore easily grabbed by the attacker’s teeth [5,8,9].

After initial stabilization the facial wounds need to be assessed for the type, size and depth of the wounds and presence of infection. Lackmann’s classification of facial bite wounds based on the extent appears in Table 1. The case described could be classified as type IIIA with some tissue loss on the labial area [10].

**Table 1:** Lackmann’s classification of facial bite injuries

**Type**	**Clinical findings**
I	Superficial injury without muscle involvement
IIA	Deep injury with muscle involvement
IIB	Full thickness injury of the cheek or lip with oral mucosal involvement (through and through wound)
IIIA	Deep injury with tissue defect (complete avulsion)
IIIB	Deep avulsive injury exposing nasal and auricular cartilages
IVA	Deep injury with severed facial nerve and/or parotid duct
IVB	Deep injury with concomitant bony fracture

The management of these injuries has undergone a transformation from allowing the wounds to heal by secondary intention to a more aggressive approach that optimizes aesthetic results by primary closure. After several years of debate there seems to be some consensus on the guidelines for the management of facial bite wounds [3]. The principle steps are as follows:

• Proper surgical toilet of the wound by irrigation

• Meticulous but not overzealous debridement of devitalized tissue

• Primary closure of the wound except in high-risk cases

• Appropriate antibiotic therapy

• Tetanus and rabies immunization where required

The current practice emphasizes that thorough irrigation of contaminated bite wounds would considerably decrease the bacterial load, remove particulate matter and reduce infection rate. Irrigation techniques range from manual irrigation using a 20 to 35mL syringe (18 to 20G needle) to pulsatile jet lavage (pressure of 50 to 70psi). Normal saline is the preferred irrigant as it does not interfere with the normal wound healing as most antiseptics do [3].

Human bite injuries carry the risk of being infected with the bacteria flora of the oral cavity and these infections are polymicrobial in nature. This often leads surgeons to consider initial debridement and delayed closure because of the fear of wound infections [7,11].

Prophylactic broad spectrum antibiotic treatment and primary closure of bite wound remain areas of controversy. Nevertheless, delayed closure should only be reserved for certain high risk or already infected wounds [3,6]. The fact that most patients have good healing of the wound after primary sutures is because of the good blood supply in the facial region which increases the host resistance [5]. Human immunodeficiency virus (HIV) transmission by human bites has been reported. Consequently, some authors have advocated routine HIV test for both victims and assailants [12,13].

Surgical debridement of devitalized tissue should be kept to a minimum in the facial area because the residual partially necrotic tissues of this anatomical region can survive on small pedicles due its rich vascularity. Landmark areas, such as the vermillion border, nasolabial fold and eyebrows should not be inadvertently trimmed [14].

The surgical reparative techniques are similar despite the aetiology of the defect. The corrective procedures in some cases are challenging and laborious. Basic principles for lip reconstruction include the remaining portions of the lip, borrowing of tissue from the opposite lip and/or the cheek, and advancement of adjacent flaps [14]. Subcutaneous sutures are kept to a minimum as they act as foreign bodies and precipitate infection. However, in avulsive wounds subcutaneous sutures may be required to get a primary closure even with the presence of a severe tissue defect [14]. The main goals of reconstruction remain the restoration of oral competence, maintenance of oral opening, and the restoration of normal anatomic relations with an acceptable aesthetic outcome [5,6].

## Conclusions

A human bite wound in the orofacial region, despite its severity and elapsed time before treatment, may have an excellent prognosis after a simple surgical procedure. At first sight, the aspect of this atypical traumatic lip injury resembled a chronic ulcerative process. A careful anamnesis and inspection will aid in characterizing the cause of any rare lip lesion.

## Consent

Written informed consent was obtained from the patient for publication of this case report and any accompanying images. A copy of the written consent is available for review by the Editor-in-Chief of this journal.

## Competing interests

The authors declare that they have no competing interests.

## Authors’ contributions

JMR was the consultant responsible for diagnosing and treating the patient in this case report. JMR provided the information to LBL, ELS and CM who co-wrote the paper. All authors have read and approved the final version of this manuscript.
